# Supplementary material for: High Burden of Hepatitis B Virus and Occult Infection Among HIV-Positive Adults and Pregnant Women in Southwest Cameroon
Source: Pathogens. 2025 Nov 5;14(11):1128. doi: 10.3390/pathogens14111128 (PMC12655005; doi:10.3390/pathogens14111128)
Supplement: Supplementary file 1 [file pathogens-14-01128-s001.zip › pathogens-3948884-supplementary.pdf]

Table S1: Primers and PCR conditions for qualitative nested PCR

<table>
<tr><td colspan="4">Primers and PCR conditions for qualitative nested PCR</td></tr>
<tr><td>PCR rounds</td><td>Primer pairs</td><td>Sequence (5′ – 3′)</td><td>PCR conditions</td></tr>
<tr><td rowspan="3">Outer</td><td>HBV-022 (sense)</td><td>TGCTGCTATGCCTCATCTTC</td><td rowspan="3">94°C for 5 minutes;
94°C for 30 seconds;
55°C for 30 seconds;
72°C for 30 seconds;
72°C for 5 minutes. (35 cycles)
Amplicon size: 408bp</td></tr>
<tr><td>HBV-065 (antisense)</td><td>CACAGATAACAAAAAATTGG</td></tr>
<tr><td>HBV-066 (antisense)</td><td>CAAAGACAAAAGAAAATTGG</td></tr>
<tr><td rowspan="3">Nested</td><td>HBV-024 (sense)</td><td>CAAGGTATGTTGCCCGTTTGTCCT</td><td rowspan="3">94°C for 5 minutes;
94°C for 30 seconds;
54°C for 30 seconds;
72°C for 30 seconds;
72°C for 5 minutes.
(35 cycles)
Amplicon size: 332bp</td></tr>
<tr><td>HBV-041 (antisense)</td><td>GGACTCAMGATGYTGCACAG</td></tr>
<tr><td>HBV-064 (antisense)</td><td>GGACTCACGATGCTGTACAG</td></tr>
<tr><td colspan="4">Primers and PCR conditions for quantitative PCR (qPCR)</td></tr>
<tr><td rowspan="3">qPCR</td><td>HBV-61</td><td>GGACCCCTGCTCGTGTTACA</td><td rowspan="3">95°C for 5 minutes;
95°C for 10 seconds;
60°C for 34 seconds;
40°C for 10 minutes.
(45 cycles)</td></tr>
<tr><td>HBV-62</td><td>GAGAGAAGTCCACCACGAGTCTAGA</td></tr>
<tr><td>HBV-TM-5</td><td>FAM5'-tgttgacaaRaatcctcacaataccRcaga-3' DabCyl</td></tr>
</table>
